# Supplementary figures and images for: Beyond the carapace: skull shape variation and morphological systematics of long-nosed armadillos (genus Dasypus)
Source: PeerJ. 2017 Aug 15;5:e3650. doi: 10.7717/peerj.3650 (PMC5562145; doi:10.7717/peerj.3650)

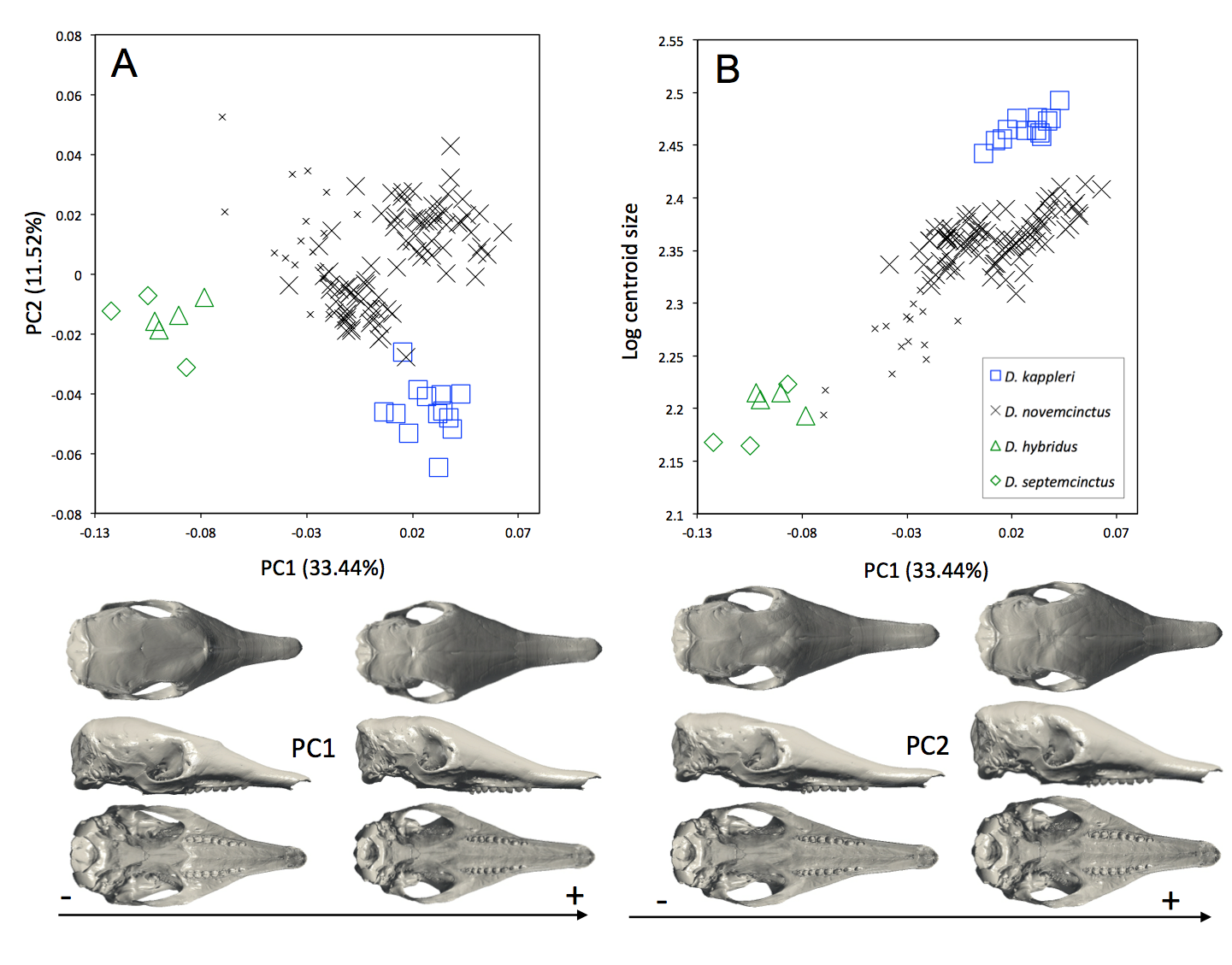

Supplement: Supplemental Information 4 — (A) Principal component analysis (PC1 vs PC2) and associated patterns of morphological transformation for crania of five Dasypus species, including juveniles (indicated with smaller symbols) and excluding D. pilosus. (B) Regression of the first principal component on the logarithm of the centroid size (R2 = 0,69; p < 0.001). Symbols: blue squares, D. kappleri; black crosses, D. novemcinctus; green triangles, D. hybridus; green diamonds, D. septemcinctus. [file peerj-05-3650-s004.png]

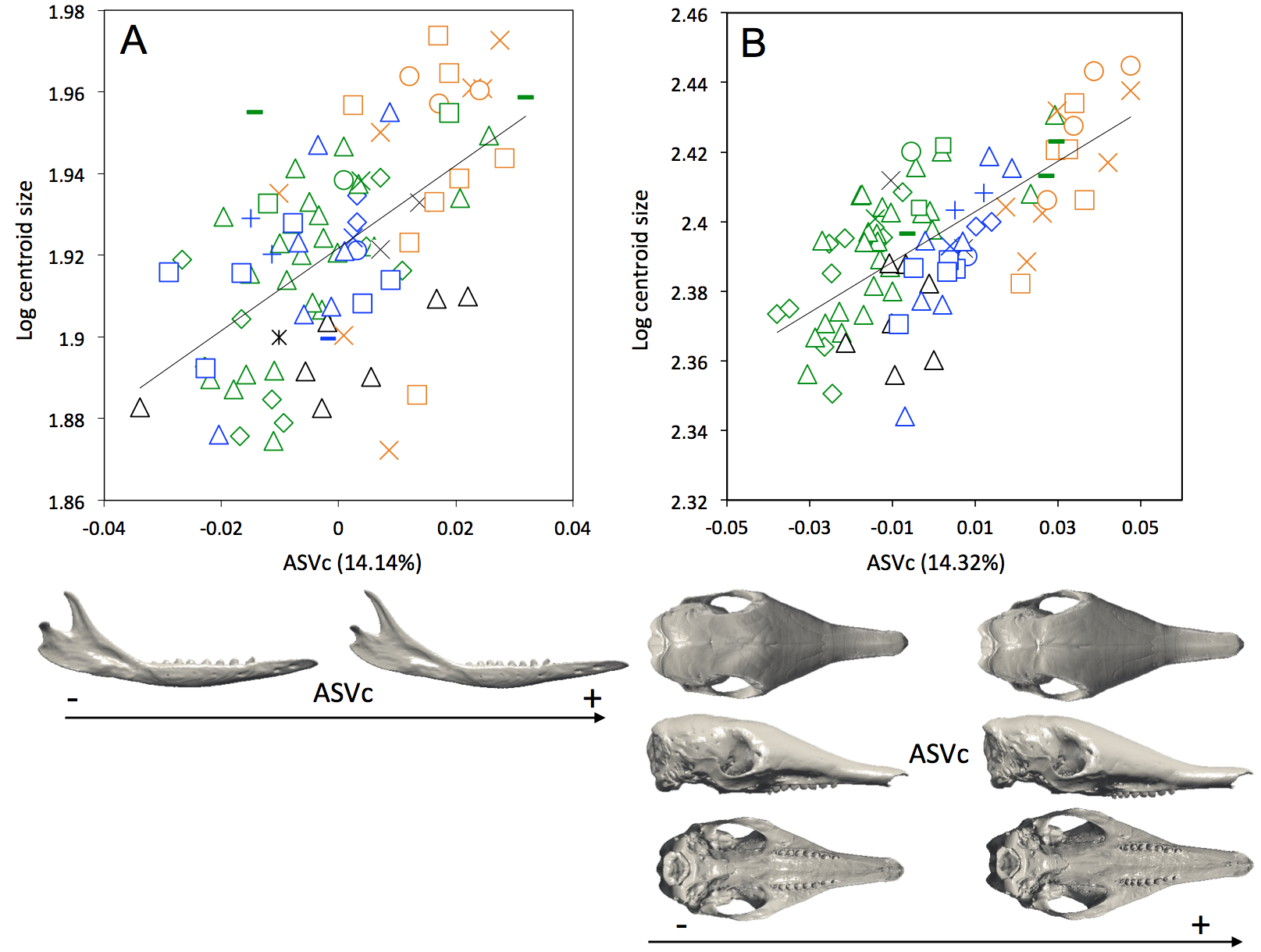

Supplement: Supplemental Information 5 — Regression of the common allometric shape vector (ASVc) on the logarithm of the centroid size for mandibles (A, R2 = 0.34; p < 0.001) and crania (B, R2 = 0 .48; p < 0.001) of D. novemcinctus. Below, associated patterns of morphological transformation for mandibles with small (left) and large (right) centroid size. Symbols: same as in Fig. 5. [file peerj-05-3650-s005.png]

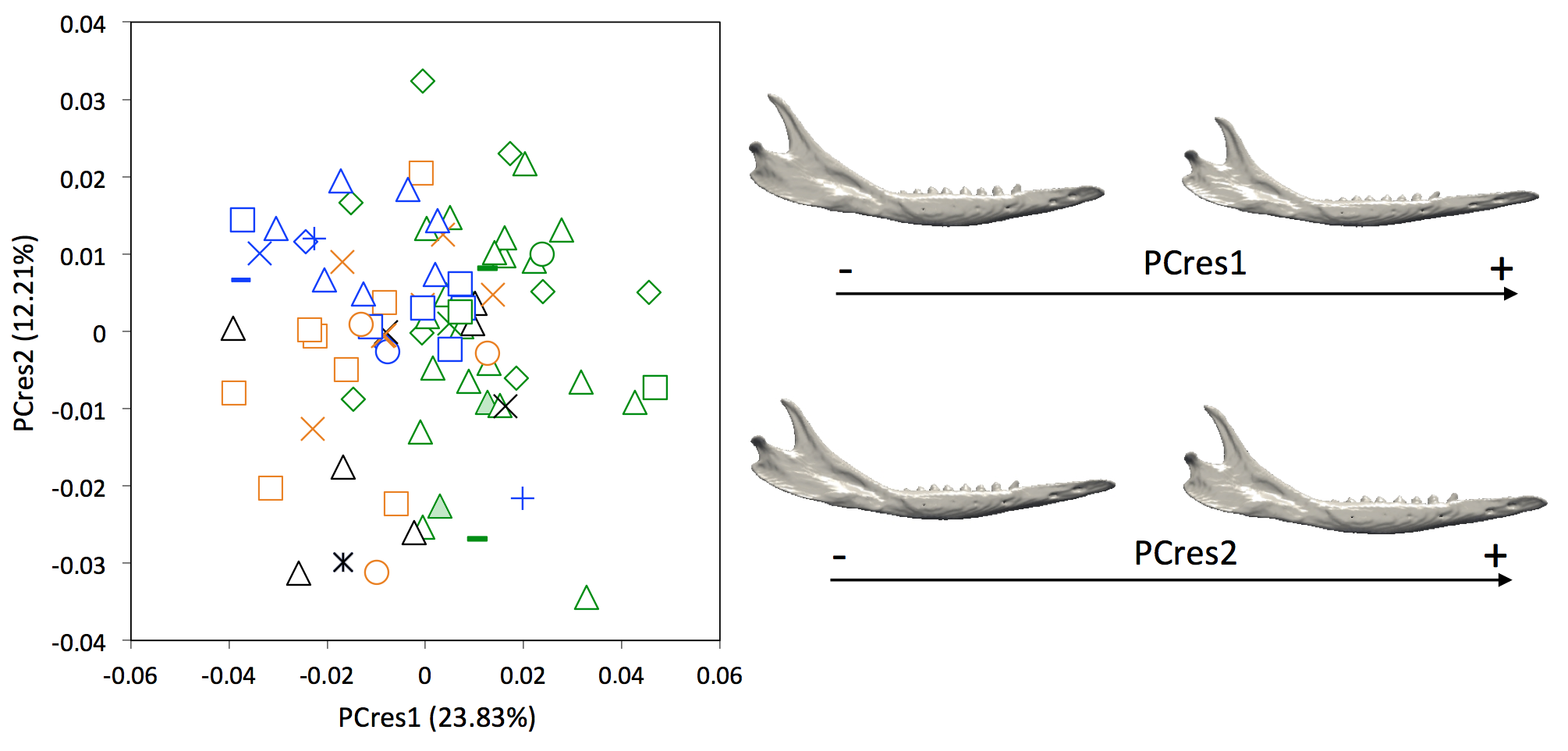

Supplement: Supplemental Information 6 — Principal component analyses with shape data corrected for allometry (PCres1 vs PCres 2) and associated patterns of morphological transformation for mandibles of Dasypus specimens. Symbols: green diamonds, Bolivia; green triangle, Brazil (solid green triangles are for specimens from Amapa); green circles, Paraguay; green crosses, Peru; green squares, Uruguay; green bars, Venezuela; blue diamonds, Belize; blue “plus,” Guatemala; blue bars, Honduras; blue squares, Mexico; blue crosses, Nicaragua; blue triangles, USA; blue circles, Costa Rica; black triangles, Colombia; black crosses, Ecuador; black stars, Panama; orange squares, French Guiana; orange crosses, Guyana; orange circles, Suriname. [file peerj-05-3650-s006.png]

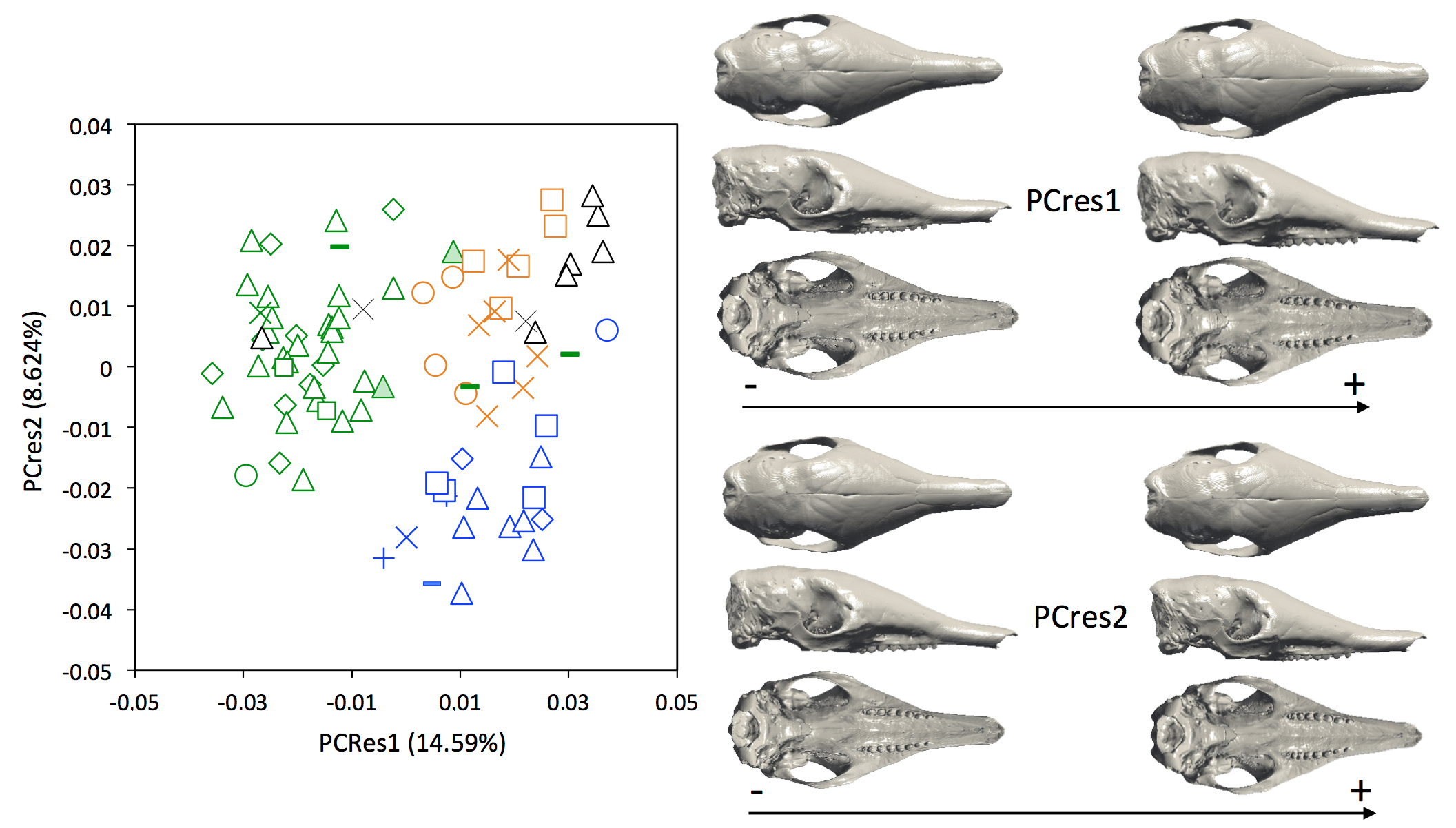

Supplement: Supplemental Information 7 — Principal component analyses with shape data corrected for allometry (PCres1 vs PCres 2) and associated patterns of morphological transformation for crania of Dasypus specimens. Symbols: green diamonds, Bolivia; green triangle, Brazil (solid green triangles are for specimens from Amapa); green circles, Paraguay; green crosses, Peru; green squares, Uruguay; green bars, Venezuela; blue diamonds, Belize; blue “plus,” Guatemala; blue bars, Honduras; blue squares, Mexico; blue crosses, Nicaragua; blue triangles, USA; blue circles, Costa Rica; black triangles, Colombia; black crosses, Ecuador; orange squares, French Guiana; orange crosses, Guyana; orange circles, Suriname. [file peerj-05-3650-s007.png]

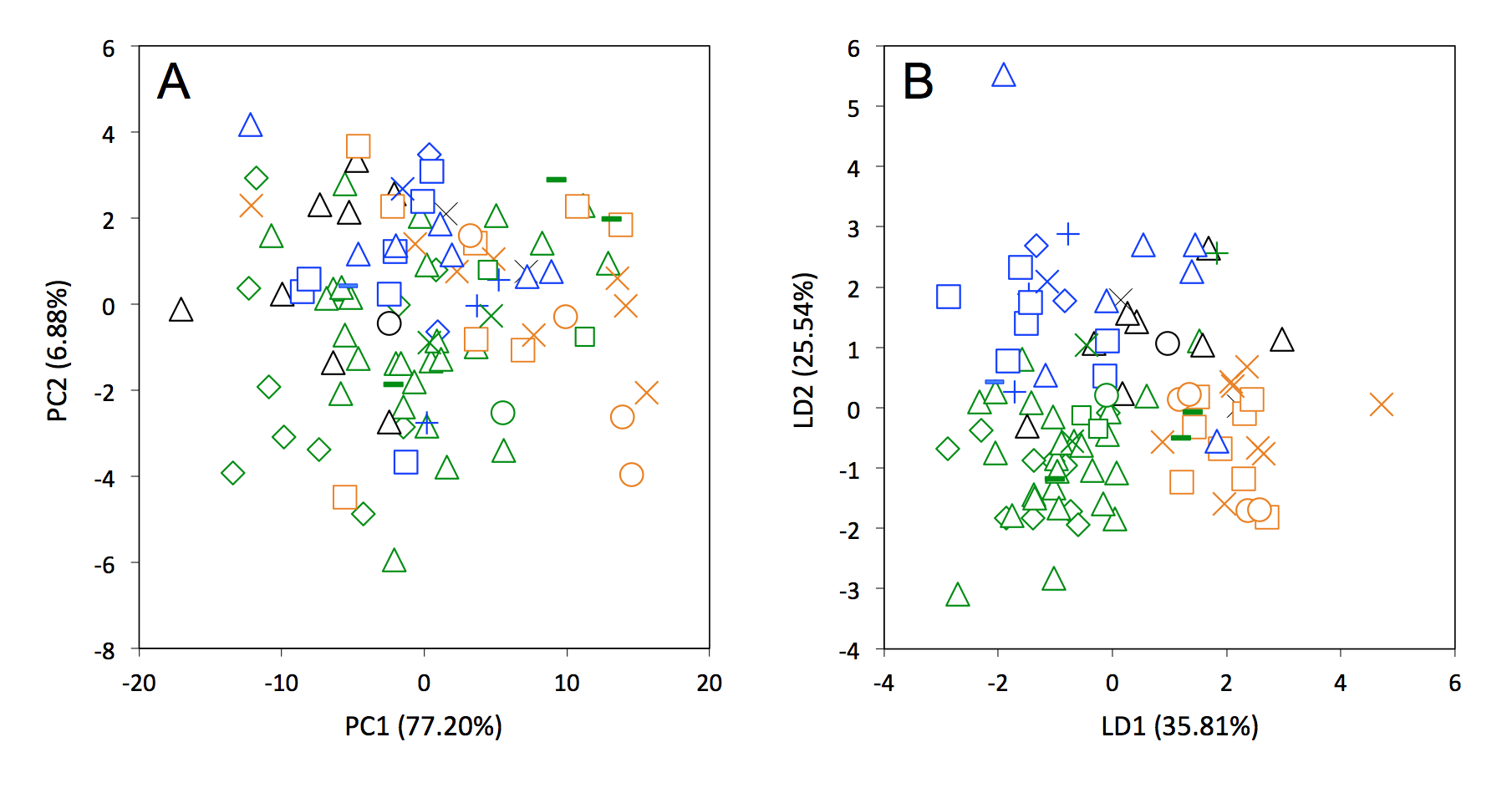

Supplement: Supplemental Information 8 — Principal component analysis (A) and linear discriminant analysis (B) performed on linear cranial measurements traditionally used in systematic studies. [file peerj-05-3650-s008.png]

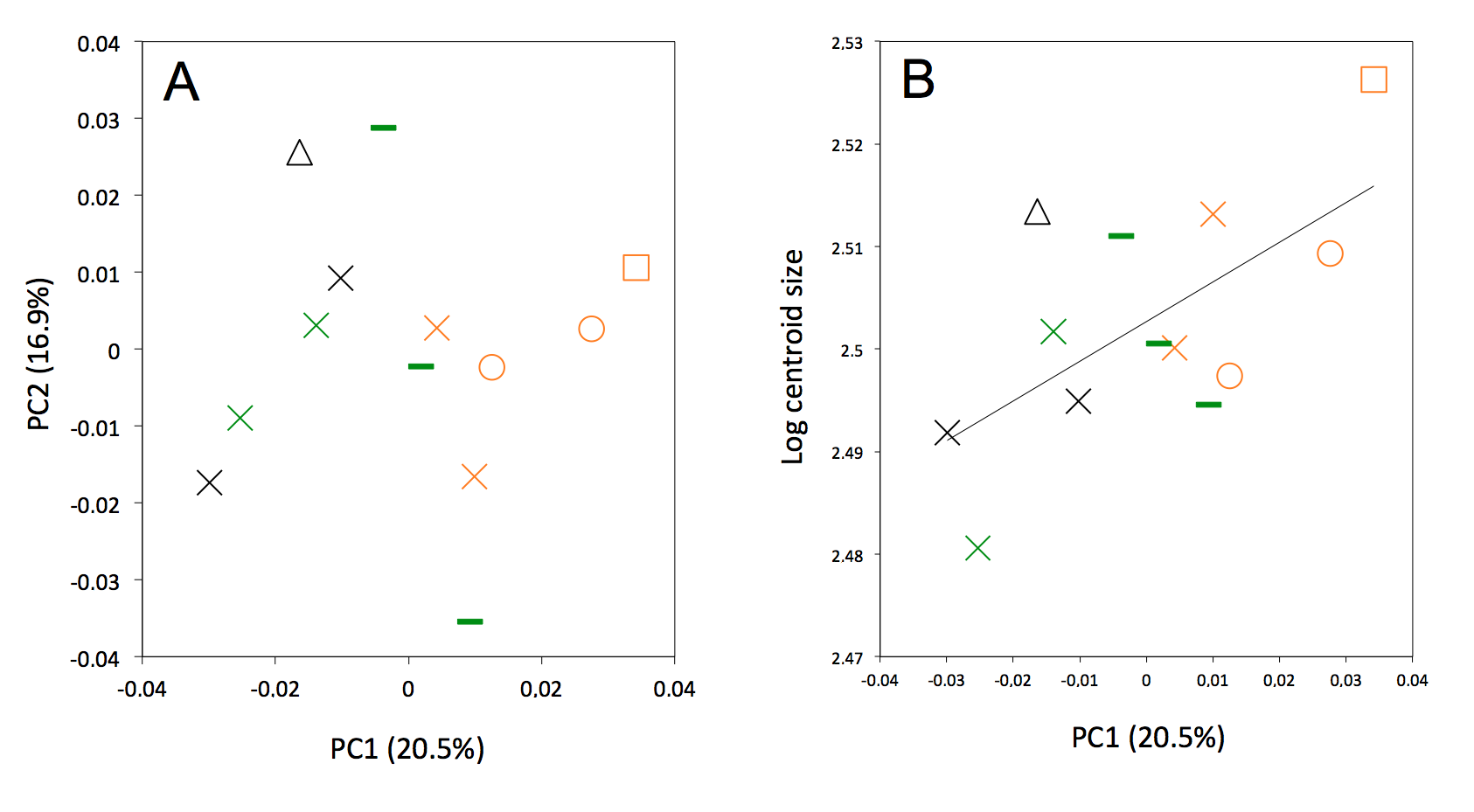

Supplement: Supplemental Information 9 — (A) Principal component analysis (PC1 vs PC2) and associate patterns of morphological transformation for crania of Dasypus kappleri. (B) Regression of the first principal component on the logarithm of the centroid size (R2 = 0.40; p < 0.001). Symbols: green crosses, Peru; green bars, Venezuela; black triangles, Colombia; black crosses, Ecuador; orange squares, French Guiana; orange crosses, Guyana; orange circles, Suriname. [file peerj-05-3650-s009.png]
